# The influence of landmark visualization style on task performance, visual attention, and spatial learning in a real-world navigation task

**DOI:** 10.1080/13875868.2024.2328099

**Published:** 2024-03-13

**Authors:** Armand Kapaj, Christopher Hilton, Sara Lanini-Maggi, Sara I. Fabrikant

**Affiliations:** a Department of Geography, University of Zurich, Zurich, Switzerland; b Digital Society Initiative, University of Zurich, Zurich, Switzerland

**Keywords:** landmark visualization, real-world navigation, spatial abilities, visual attention, spatial learning

## Abstract

Depicting landmarks on mobile maps is an increasingly popular countermeasure to the negative effect that navigation aids have on spatial learning – landmarks guide visual attention and facilitate map-to-environment information matching. However, the most effective method to visualize landmarks on mobile map aids remains an open question. We conducted a real-world navigation study outdoors to evaluate the influence of realistic vs. abstract 3D landmark visualization styles on wayfinders’ navigation performance, visual attention, and spatial learning. While navigating with realistic landmarks, low-spatial-ability wayfinders focused more on the landmarks in the environment and demonstrated improved knowledge of directions between landmarks. Our findings emphasize the importance of visual realism when enriching navigation aids with landmarks to guide attention and enhance spatial learning for low-spatial-ability wayfinders.

## 1. Introduction

Imagine you are meeting friends at a café in an unfamiliar area of the city. As you do not know the area and live in a spatially enabled society (Ishikawa, 2019), you will most likely rely on your trusted, ready-installed mobile map on your smart assistive device to help you reach the desired destination (Dahmani & Bohbot, 2020). At some of the busy intersections on your route with poor GPS localization, finding your current location and determining your orientation with the mobile map is difficult. At these moments, you visually scan the environment and the mobile map and try to match the visual information experienced directly in the real world with the graphic representation on the navigation aid. Eventually, you hopefully manage to reach the desired café. How much were you able to learn about your newly traversed part of the city? Would you have learned more if salient, navigation-relevant features of the traversed environment had been visualized on the map with greater detail? Would greater fidelity of the visualization have guided your visual attention to important information more directly, and would your journey have been quicker and less error-prone as a result? These are the questions addressed in this empirical mobile map-aided navigation study.

Humans use their locomotion skills to complete everyday wayfinding tasks in familiar and unfamiliar environments (Montello, 2005). During wayfinding, humans continuously acquire spatial knowledge of the traversed environment (Montello, 1998, 2005), consisting of landmark, route, and survey knowledge (Siegel & White, 1975). Landmarks are geographic objects that stand out from their surroundings due to their visibility and saliency (Richter & Winter, 2014), and landmark knowledge refers to memory for those objects. Route knowledge is the binding of landmarks into sequences and the associated turning directions at landmarks for reaching a specific destination (Hirtle & Hudson, 1991; Montello, 1998; Siegel & White, 1975). Survey knowledge refers to the integration of routes and landmarks into a common frame of reference that incorporates metric information, allowing for goal-independent, flexible navigation (Montello, 1998; Siegel & White, 1975). Development of the different representation types and navigation ability, in general, is also influenced by individual spatial abilities and familiarity with the given environment (Hegarty et al., 2006; Ishikawa, 2023; Montello, 1998; Newcombe, 2018; Newcombe et al., 2022; Woollett et al., 2009).

When it comes to varied map-aided navigation tasks (see Wiener et al., 2009 for a taxonomy of navigation tasks), the resulting spatial learning process is altered in different ways (Dahmani & Bohbot, 2020). Past empirical research has demonstrated that over-reliance on mobile maps negatively influences wayfinders’ spatial knowledge acquisition of the traversed environment in the short term and further contributes to the long-term deterioration of their spatial abilities (Dahmani & Bohbot, 2020; Ishikawa, 2019; Ruginski et al., 2019). The negative effects of mobile maps are primarily attributed to changes in wayfinders’ visual behavior (Gardony et al., 2013, 2015; Hejtmánek et al., 2018) and to users entering a passive navigation state where the task of navigation is delegated to the map aid (Chrastil & Warren, 2012; Ishikawa, 2019; Willis et al., 2009). The modulation of visual attention during aided navigation leads to a divided and selective attention between the mobile map display and the environment (Gardony et al., 2013, 2015; Hejtmánek et al., 2018; Willis et al., 2009). Consequently, the rate of spatial knowledge acquisition declines as the mobile map aid reduces the allocation of attentional resources to task-relevant environmental features that are important for spatial learning (Chrastil & Warren, 2012; Gardony et al., 2013, 2015; Hejtmánek et al., 2018; Willis et al., 2009).

A suggested core factor in the mobile map-aided navigation context is the lack of expressively and visually communicated landmark information. It is already well-established that landmarks are important for spatial learning because they serve as anchors and reference points that help wayfinders easily orient themselves in the surrounding environment, navigate to a destination beyond their immediate surroundings, and structure their mental representation of space (Couclelis et al., 1987; Golledge, 1999; Montello, 2005). Empirical navigation studies have already provided evidence that wayfinders’ ability to learn unfamiliar environments is significantly better when such environments are enriched with landmarks compared to when no landmark information is available (Cliburn et al., 2007; Heft, 1979; Jansen-Osmann & Fuchs, 2006; Sharma et al., 2017; Waller & Lippa, 2007). Such navigation improvements occur alongside a fundamental shift in the dynamics of visual attention, where landmarks in the environment that are prioritized by a navigator’s visual encoding strategies are directly related to their importance in the resulting spatial representation (Hamid et al., 2010). It can also be argued that landmarks could facilitate self-localization and orientation processes in aided navigation by allowing the visual matching of the allocentric birds-eye-view of the mobile map with the egocentric first-person perspective experienced during active navigation (Chrastil & Warren, 2012; Kiefer et al., 2014; Montello, 2005; Richter & Winter, 2014; Willis et al., 2009).

Despite their acknowledged contribution to various components of spatial navigation and human cognition (see reviews by Chan et al., 2012; Epstein & Vass, 2014; Epstein et al., 2017; Yesiltepe et al., 2021), landmarks are not communicated effectively on mobile navigation systems to support wayfinders’ spatial learning (Richter & Winter, 2014). Current mobile navigation systems often omit landmarks entirely or depict them only as abstract 2D building footprints with no further visual detail and in a similar style to other buildings. By now, it is a common idea that landmarks on mobile maps can aid the guidance of attention toward the important parts of the environment, thus counteracting the negative changes in the direction of visual attention that are implicated in map-related spatial ability degradation (Chrastil & Warren, 2012; Richter & Winter, 2014; Willis et al., 2009). So, whilst the benefit of landmark information for efficient navigation is clear, the means by which to effectively communicate that information on mobile navigation systems have yet to be established (Richter & Winter, 2014).

**Figure 1.**
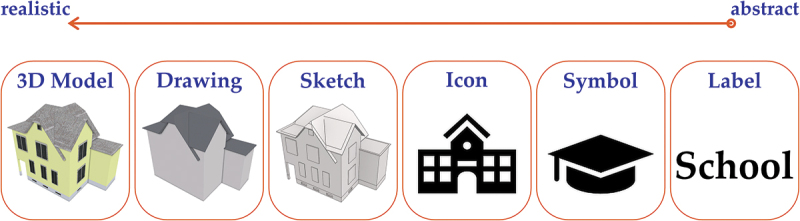
Graphical abstraction continuum for visualizing point feature landmarks on mobile maps (after Elias & Paelke, 2008, p. 44).

From a cartographic design perspective, landmarks can be depicted on mobile maps on a graphical continuum ranging from abstract 2D symbols to realistic 3D buildings (Elias & Paelke, 2008; MacEachren, 2004; Montello et al., 2018). Based on this, Elias and Paelke (2008) proposed a cartographic design concept for depicting point features (i.e., buildings) as landmarks on mobile maps along a graphical abstraction continuum ranging from abstract 2D labels to realistic 3D buildings (Figure 1). The authors proposed that in order to retain building names or types, they can be visualized as labels (e.g., Starbucks), symbols (e.g., coffee cup symbol), or icons (e.g., a trademark logo); however, the authors recommended that buildings with a characteristic architectural style, salient facade, specific function, or a prominent location should be depicted using realistically textured 3D models, 3D drawings of the building outline, or at least a 3D sketch of the building structure including additional information of characteristic building elements (e.g., windows, doors, etc.) to more easily retain their visual properties and to facilitate the visual matching with referenced buildings in the real world (cf. MacEachren, 2004, p. 259).

Even though the cartographic design guidelines of Elias and Paelke (2008) were not assessed in a navigation context, they constitute adequate means for retaining the visual information of landmarks, such as color and texture, on mobile maps. The presence of landmarks as visuospatial cues on navigation map aids is essential for spatial knowledge formation (Ekstrom, 2015; Epstein & Vass, 2014; Ramanoël et al., 2022). Therefore, visualizing navigation-relevant landmarks on mobile maps as 3D buildings with higher visual fidelity compared to less relevant features provides a pedestrian navigator with a more naturalistic depiction of the experienced environment, which in turn facilitates feature identification between the physical world and the mobile map display, and spatial learning (Chrastil & Warren, 2012; Raubal & Winter, 2002; Richter & Winter, 2014; Thrash et al., 2019; Willis et al., 2009). Additionally, users generally prefer fully realistic 3D visualizations, which is attributed to the fact that they provide a more naturalistic representation of the environment and are perceived as recognizable, efficient, intuitive, enjoyable, and fun (Hegarty et al., 2009; Liao et al., 2017; Lokka & Çöltekin, 2019; Oulasvirta et al., 2009; Plesa & Cartwright, 2008). However, even though users might prefer fully realistic-looking 3D visualizations, they do not necessarily perform better with these design solutions (Franke & Schweikart, 2017; Hegarty et al., 2009; Kapaj et al., 2021; Kray et al., 2003; Liao et al., 2017; Oulasvirta et al., 2009; Plesa & Cartwright, 2008). Indeed, studies implementing more complex designs into navigation aids have suggested that too much visual information can unnecessarily increase decision times and memory load (Hegarty et al., 2009, 2012; Lei et al., 2016; Liao et al., 2017; Lokka & Çöltekin, 2019; Plesa & Cartwright, 2008).

Some researchers have proposed a depiction of only task-relevant landmarks as realistic 3D models on a 2D basemap (Kapaj et al., 2021; Lei et al., 2016; Liao et al., 2017). As attention is attracted by items that differ from their surroundings (Wolfe & Horowitz, 2017), depicting landmarks as realistic 3D building models on a 2D basemap serves as a bottom-up perceptual guidance by stimulus salience, guiding users’ attention to these features (Wolfe & Horowitz, 2017). Consequently, the system is improved in two steps: visual attention is organically guided toward salient, task-relevant landmarks on the map display (Richter & Winter, 2014; Wenczel et al., 2017), and the visual matching of those landmarks in the real environment and the mobile map is facilitated by their realistic visualization (Kiefer et al., 2014; Liao et al., 2017; Richter & Winter, 2014). Therefore, these suggested improvements may not only help to enhance usability and wayfinding efficiency but also mitigate the negative effects of navigation aid use (Dahmani & Bohbot, 2020; Ishikawa, 2019; Ruginski et al., 2019) and divided attention on spatial learning (Gardony et al., 2013, 2015; Hejtmánek et al., 2018; Kapaj et al., 2021) through a reduced mismatch between real and displayed landmarks.

Thus, the state of research into map landmark visualization indicates that a 2D base map with 3D landmark models provides a sound cartographic design choice for retaining the graphic characteristics of a task-relevant object (MacEachren, 2004, p. 259) and combining the benefits of both representation styles (Lei et al., 2016; Liao et al., 2017). However, the visual fidelity of the landmark models has yet to be properly assessed. Some work argues for abstract 3D models as they provide the necessary visual information for facilitated wayfinding. For instance, Plesa and Cartwright (2008) conclude that an abstract 3D visualization – similar to the 3D sketch design recommendation of Elias and Paelke (2008) – provides users with the necessary visual information for effective wayfinding. However, in Plesa and Cartwright’s (2008) study, many features of the environment, including those not relevant for navigation, were manipulated to be visualized in realistic or abstract 3D styles. Therefore, they did not directly compare the *landmark* visualization styles, and their findings could have been influenced by non-landmark features such as the aforementioned information overload that arises from a 3D realistic style on the whole map. Indeed, their conclusion that landmark visualizations with lower levels of realism are preferable is in contrast with the idea that loss of visual fidelity in landmarks would increase the mismatch between mobile map display and environment, which is argued as a defining benefit of landmark visualization in the first place (Kiefer et al., 2014; Liao et al., 2017; Lokka & Çöltekin, 2019; Richter & Winter, 2014).

In a recent study, Ramanoël et al. (2022) investigated navigation performance and the neural networks associated with navigating a virtual “Y” maze environment using abstract 3D objects and textured or colored 3D features (i.e., walls). The navigation performance results revealed no differences in completion time and success rates across the object and feature conditions, indicating that they provide equivalent visuospatial cues to facilitate orientation in space (Ramanoël et al., 2022). The object-based condition elicited an enhanced and widespread activation pattern in the temporal and occipital lobes compared to the feature-based condition. The authors speculate that integrating abstract 3D objects for spatial navigation requires detailed visual processing that is unnecessary for the textured or colored 3D features, thus requiring more cognitive processes for efficient spatial navigation. However, again, this study did not examine different visualization styles of landmark objects, but rather just different architectural features of the environment, and so their findings that more detailed textures reduced cognitive demands during navigation may also apply to landmark objects. Ramanoël et al. (2022) suggested that future studies should utilize eye-tracking methods to investigate whether landmark visual properties modulate wayfinders’ visual attention behavior and how it predicts navigation and spatial learning performance.

Overall, existing research shows that the realism of environmental information affects navigation and spatial learning processes, but so far, no direct comparison of individual landmarks in different styles has been made, nor has it been applied to mobile map design for map-assisted navigation in the outdoors. Therefore, we conducted a real-world route following navigation study to assess how different landmark visualization styles might interact with wayfinders’ landmark, route, and survey knowledge (Montello, 1998; Siegel & White, 1975). To mitigate visual clutter (Rosenholtz et al., 2007) on the mobile map but to direct wayfinders’ visual attention (Wolfe & Horowitz, 2017) to task-relevant landmarks so that they will attend to the environment and engage in spatial learning (Chrastil & Warren, 2012; Gardony et al., 2013, 2015; Hejtmánek et al., 2018; Ishikawa, 2019; Kapaj et al., 2021; Willis et al., 2009), we visualized navigation-relevant landmarks as 3D buildings on a 2D basemap (Lei et al., 2016; Liao et al., 2017). The 3D landmarks were depicted as either realistic (textured) or abstract buildings to assess whether graphic realism affects performance due to added visual information (Hegarty et al., 2009, 2012; Lei et al., 2016; Liao et al., 2017; Plesa & Cartwright, 2008). We were interested in examining how different landmark visualization styles (i.e., abstract 3D buildings versus realistic 3D buildings) might influence participants’ navigation performance, gaze behavior, and spatial learning during a real-world navigation task. In addition, we assessed how participants’ visual attention influenced their acquired knowledge of the environment. Particularly, we sought to answer the following research questions (RQ) and associated hypothesis (H):

RQ1: What are the differences in participants’ navigation performance, visual attention, and landmark, route, and survey knowledge acquisition of the traversed environment while navigating a real-world environment with the aid of a mobile map depicting landmarks as abstract 3D or realistic 3D building models?

We hypothesized (H1) that participants will visually attend to the environment and the navigation-relevant landmarks in it for a longer period and thus less to the mobile map display when navigating with realistic 3D landmarks. This is because of the greater visual saliency of the landmarks on the mobile map. The longer visual attention on navigation-relevant landmarks will facilitate the visual matching of the information on the mobile map display with respective information in the environment (Kapaj et al., 2021; Kiefer et al., 2014), in turn, leading to better navigation performance, including improved landmark, route, and survey knowledge acquisition.

RQ2: How does the distribution of participants’ visual attention during navigation influence their landmark, route, and survey knowledge acquisition?

We hypothesized (H2) that navigators’ longer visual attention in the environment and on the navigation-relevant landmarks and, thus, shorter visual attention on the mobile map will lead to improved landmark, route, and survey knowledge. This is because higher attention on the mobile map instead of the environment will disengage wayfinders with the traversed environment, leading to missed task-relevant environmental cues necessary for spatial learning (Chrastil & Warren, 2012; Gardony et al., 2013, 2015; Hejtmánek et al., 2018; Kapaj et al., 2023; Koletsis et al., 2017; Willis et al., 2009).

The in-situ, real-world assessment of mobile-map-aided pedestrian navigation can contribute ecologically valid new insights into how different landmark visualization styles can influence wayfinders’ landmark, route, and survey knowledge acquisition (Ishikawa & Montello, 2006; Ishikawa et al., 2008; Kiefer et al., 2014; Münzer et al., 2006).

## 2. Methods

### 2.1. Participants

We conducted an a-priori power analysis for a single-predictor multilevel linear regression model (DeBruine & Barr, 2021) using the *simr* package (Green et al., 2016) for data simulation in R (v.4.2.1). Using parameters estimated from own previous study (i.e., fixed effects beta coefficients and random effects’ variance; Kapaj et al., 2023), we found that a minimum of 40 participants, each completing 10 pointing task trials would achieve an 83% power (see data availability statement for study preregistration).

In total, 46 healthy adults participated in the experiment, including 22 females (average age = 27.3 years, range = 21–46 years) and 24 males (average age = 27.8 years, range = 22–38 years). Ten participants rated themselves as having some familiarity with the study area, and the remaining 36 rated themselves as unfamiliar. Since familiarity with the study area might influence wayfinders’ spatial learning (Montello, 1998; Siegel & White, 1975), we controlled for the effect of familiarity in our analyses. The ethical approval for this study was provided by the Ethics Committee of the University of Zurich (no. 19.6.10), and participants gave written informed consent before the start of the experiment. Participation criteria were normal or corrected to normal vision (only contact lenses as eyeglasses would interfere with eye-tracking glasses) and no history of physical and psychiatric disorders that may interfere with the navigation task. The experiment lasted approximately two hours, and participants were compensated with 40 Swiss Francs upon completing the experiment.

### 2.2. Route following navigation task

In the learning phase, participants had to navigate a residential urban area in Zurich, Switzerland, aided by a mobile map. Participants were asked to follow a predefined route depicted on the mobile map application and identify the 10 landmarks visualized on the mobile map (i.e., abstract or realistic 3D buildings) in the environment by raising their hand while next to the landmark and proceeding to reach the destination. Participants were instructed to walk as they would when navigating a new environment. In addition, we instructed the participants that their knowledge of the environment would be assessed after the navigation task – thus testing their intentional spatial learning performance (Chrastil & Warren, 2012) – without revealing the exact nature of the spatial knowledge tests that would be utilized. During the task, participants were shadowed at a safe distance by the experimenters, who recorded their navigation performance (i.e., correct turns and identifying relevant landmarks).

### 2.3. Experimental design

We used a within-subject experimental design with the landmark visualization style on the mobile map (abstract 3D vs. realistic 3D building models) as the independent variable. Several dependent variables were recorded in this study: navigation accuracy and completion time, visual attention (i.e., eye-movement recordings during navigation), recognition of relevant landmarks and associated route directions, reconstruction of the landmark sequence, and survey knowledge acquisition tasks. We also recorded participants’ interactions with the mobile map, self-reported task workload, and objectively measured cognitive load with electroencephalography (EEG). However, results on map interactions and cognitive load are not presented in this paper, which focuses on visual attention and spatial learning and thus will only be mentioned as part of the study procedure for replicability.

### 2.4. Materials and apparatus

#### 2.4.1. Navigation phase route

**Figure 2.**
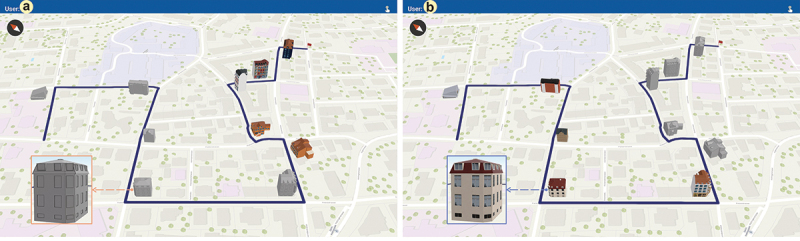
The interactive mobile map application depicts navigation-relevant landmarks either as abstract (a) or realistic (b) 3D buildings (the inset view shows a zoomed-in landmark in the respective visualization style) and the route to be followed.

For the route-following portion of the experiment, participants were asked to follow a predefined route depicted as a blue line on an interactive mobile map (Figure 2) to serve participants as a navigation aid we specifically developed for this study. The route was approximately 1 km, containing five right, four left turns, and one straight movement at intersections. We selected ten buildings as navigation-relevant landmarks, that is, one building per intersection, and visualized these as either abstract or realistic 3D buildings on the mobile map. We counterbalanced the order of the landmark visualization style, starting with the first five landmarks depicted as either abstract (Figure 2a) or realistic (Figure 2b) buildings on the mobile map.

To inform the selection of landmarks to display on the mobile map, we conducted a small survey (*N* = 9) prior to the navigation study to select one building per intersection that would serve as a landmark. We provided participants in this pilot study with a sketch map of the intersection, their position approaching the intersection, moving direction, and photographs of the buildings – as seen from the participants’ viewpoint – indicating their location on the intersection. We asked participants to rate the most prominent building or the building they would use when giving directions or the building that is the easiest to describe (Nothegger et al., 2004).

We used CityEngine 2019.0 (Esri, CA, USA; https://www.esri.com/en-us/arcgis/products/arcgis-cityengine) to build the 3D buildings, and ArcGIS Pro 2.8 (Esri, CA, USA; https://www.esri.com/en-us/arcgis/products/arcgis-pro) to design the mobile map stimuli. To texturize the realistic 3D building models, we took photos of landmarks in similar weather and lighting conditions to control for appearance issues (Winter et al., 2005). The stimuli were developed as interactive mobile map applications using the ArcGIS API for Java (Esri, CA, USA; https://developers.arcgis.com/java/) and deployed on a 10.1″ SAMSUNG Galaxy tablet with 1920 by 1200 display resolution. Participants could interact with the mobile map display (e.g., zoom, pan, rotate, and tilt) as desired.

#### 2.4.2. Sensory recordings and questionnaire measures

**Mobile eye-tracking** (MET) glasses allowing for unrestrained head movements were employed to record participants’ gaze behavior during navigation. We used Pupil Invisible, a binocular head-mounted mobile eye-tracking glasses from Pupil Labs (Pupil Labs, Berlin, Germany; https://pupil-labs.com/products/invisible/). Pupil Invisible, a video-based pupil-corneal reflection eye tracker, does not require calibration and offers a gaze precision accuracy of 4° for real-world studies, independent of lighting conditions and participant-specific factors (e.g., age, gender, contact lenses, interpupillary distance, and makeup; Tonsen et al., 2020). Eye movements are recorded in a companion app and uploaded to Pupil Cloud at a sampling rate of 200 Hz. The MET glasses contain a scene camera with 1088 by 1088 resolution and an 82° by 82° field of view, which records at 30 Hz. We used shaded or clear lenses to prevent participants from squinting depending on weather conditions. The MET glasses were connected to an accompanying mobile device for data recording, which participants carried in a backpack.

**Electroencephalography** (EEG) and **NASA Task Load Index** (NASA TLX; Hart & Staveland, 1988) were recorded to address a different research question beyond the scope of the present study and thus will not be mentioned further.

**Questionnaire on spatial strategies** (QSS) is a standardized instrument that contributes to explaining individual differences in spatial abilities related to environmental navigation (Münzer & Hölscher, 2011; Münzer et al., 2016). The QSS is a 19-item questionnaire where participants self-report on a 7-point Likert scale how much they rely on landmark, route, direction, or mental map strategies in various environments (i.e., familiar, unfamiliar, indoor, and outdoor environments). The questions are divided into three spatial strategies scales: 1) the global-egocentric orientation scale, including the sense of direction, aimed to capture participants’ general ability and egocentric strategies based on route and direction knowledge; 2) the survey scale to capture participants’ mental map knowledge; and 3) the cardinal directions scale to capture knowledge of cardinal directions (Münzer & Hölscher, 2011; Münzer et al., 2016). The QSS has been found to predict wayfinders’ spatial learning and orientation in real-world environments and shows incremental validity over relevant predictors of wayfinders’ cognitive visuospatial abilities (Münzer & Hölscher, 2011). We computed the average score of the 19 questions to generate an overall spatial ability score to assess how this self-reported spatial ability score influences participants’ spatial learning. Additionally, we collected participants’ demographic data (such as gender, age, and profession) as part of the QSS questionnaire.

**Perspective taking and spatial orientation test** (PTSOT, Hegarty & Waller, 2004; Kozhevnikov & Hegarty, 2001) was used to assess participants’ abilities to imagine different perspectives and orientations in space. The PTSOT uses an array of objects in 12 trials and asks participants to imagine standing at one object, facing another, and indicating the direction of a third object. The task was conducted as described by Hegarty and Waller (2004) and Friedman et al. (2020), including the exclusion of one participant due to their error being higher than chance performance.

**Landmark and route knowledge questionnaire** assessed participants’ landmark recognition (i.e., landmark knowledge) and memory for associated route directions (i.e., route knowledge). The task was conducted following its successful implementation by Wunderlich et al. (2022) and Wunderlich and Gramann (2021). Specifically, participants were shown an image of a building to which they first indicated whether they recognized the building as being from the learned route or not. If they did recognize the building, they were asked to indicate whether it was depicted on the mobile map in 3D and to recall the direction of travel taken at that building. There were 10 such landmarks from relevant decision point intersections that were depicted on the map (referred to as REL), 10 landmarks considered task-irrelevant but encountered on route segments without any decision points and not depicted on the mobile map (referred to as IRL), and 10 additional, novel buildings from elsewhere in the study area that were not encountered along the route nor featured on the map (referred to as NOV), for a total of 30 trials.

**Landmark free reconstruction of order task** (Hilton et al., 2021) assessed whether participants could place the 10 REL landmarks in the correct sequence as they were encountered along the navigated route to assess their acquired route knowledge further. We provided participants with printout images of all the buildings at once, as seen from their perspective during the navigation task, and participants had to write down the sequence they believed they encountered the building during the task. Participants had no time constraints and were free to move the printouts in any order they wished and could correct their answers as often as they wanted.

**Direction and distance estimation task** was used to assess participants’ survey knowledge acquisition of the traversed environment only for the REL landmarks. The task was conducted as in Ishikawa and Montello (2006). On A4 paper, we showed images of two landmarks with their names and a 10 cm radius circle with the name of one of the landmark pairs in the center. Participants were asked to imagine being at a particular landmark, facing straight ahead as when they had learned the landmark walking along the navigation route, and to draw a line from the center of the circle to indicate the direction of the target landmark constituting the pair. Additionally, we asked participants to indicate the straight-line distance between the pairs for the distance estimation task in meters. As we wished to assess the influence of landmark visualization styles on wayfinders’ survey knowledge, we did not ask participants to estimate the direction and distance of depicted landmarks across the visualization conditions. Testing only within a visualization condition (i.e., abstract or realistic) resulted in 10 landmark pairs out of 20 possible permutations per condition (5 × 4), which were randomly chosen per participant.

### 2.5. Experimental procedure

The experiment was conducted in English on days with suitable weather conditions. Participants were welcomed to a preparation room close to the study area, where they received instructions and completed the informed consent procedure, the PTSOT, the QSS, and the demographic questionnaire, including where the EEG cap and eye-tracking device were set up.

We led participants to the start of the route shown on the mobile map to be followed and asked them to get familiar with the tablet device and how to interact with it. We shadowed participants along the route following task and recorded their navigation performance (i.e., correct turns and landmark identification). If the participants made an error during navigation, the experimenter called them back to the intersection where the wrong navigation decision was made and prompted them to continue. After the navigation task, we removed the EEG cap and MET glasses and guided participants back to the preparation room. The time from the end of the navigation task to the start of the follow-up tests was approximately 10 minutes and included a public transport trip. Participants completed the NASA TLX to rate their perceived navigation task workload during the trip back. After arriving at the preparation room, participants completed the landmark and route knowledge task, the landmark free reconstruction of order, and the landmark direction and distance estimation task.

### 2.6. MET data processing

On average, overall, 16.7% of the raw gaze samples were lost (*M* = 16.7, *SD* = 4.31), 16.6% of which were due to participant blinks (*M* = 16.6, *SD* = 4.34), amounting to an eye-movement tracking ratio of 99.8% (*M* = 99.8, *SD* = 0.10). We annotated participants’ raw gaze data using the iMotions (iMotions, Copenhagen, Denmark; https://imotions.com/) platform. We categorized participants’ eye fixation data into three areas of interest (AOI). The first AOI corresponds to the “environment” (i.e., environmental features except for the 10 landmark buildings), the second AOI to the “mobile map” display, and the third is the “landmark” AOI (i.e., the 10 task-relevant buildings). Raw gaze data were subsequently parsed into fixation and saccades in MATLAB using the EYE-EEG plugin (v0.99; Dimigen et al., 2011) for the EEGLAB toolbox (v2022.1; Delorme & Makeig, 2004), and these fixations received the AOI label of the underlying raw gaze samples. The velocity threshold for a saccadic eye movement was defined at 6 SD above the participants’ median gaze velocity for at least four consecutive samples (Engbert & Kliegl, 2003; Engbert & Mergenthaler, 2006). Fixations can vary greatly in duration – as short as 30–40 ms and as long as over 2000 ms (Holmqvist et al., 2011, p. 381; Velichkovsky et al., 2019) – and convey different cognitive processes (Holmqvist et al., 2011, p. 416; Schleicher et al., 2008). According to Schleicher et al. (2008), fixations shorter than 150 ms, or so-called express fixations, are caused by low-level visuomotor behavior and are insufficient to extract relevant information about cognitive processes. We thus discarded fixations shorter than 150 ms because we wished to capture cognitive processes and removed fixations longer than 2000 ms as a standard cutoff threshold to remove fixation outliers (e.g., Brockmole & Henderson, 2008; Cornelissen & Võ, 2017). The remaining fixations on the above-defined AOIs were summed and normalized by wayfinders’ trial completion time (as a percentage), using the equation below:

(1) $$\mathrm{nFD}\;=\;\frac{\mathrm{Sum}\;\;of\;\;f\mathrm{ixation}\;\;d\mathrm{urations}\;\;on\;\;AOI\;\;\left[\mathrm{ms}\right]}{\mathrm{Trial}\;\;t\mathrm{ime}\;\left[\mathrm{ms}\right]}\;\times \;100$$

### 2.7. Data analysis

To examine the effect of visualization style on participants’ landmark knowledge, we ran analyses based on signal detection theory (SDT; Tanner & Swets, 1954) using the *psycho* package (Makowski, 2018) in R (v.4.2.1). We used the *discriminability index d prime* [*d’* = z(hit rate) – z(false alarm rate)] to compare landmarks recognition accuracy between the visualization conditions. We used SDT to run a two-fold analysis. First, we analyzed recognition memory for landmarks present in the environment (i.e., REL and IRL) versus those not in the environment (i.e., NOL) during the navigation task. Second, we analyzed recognition memory for landmarks present in the environment and depicted on the mobile map (i.e., REL) versus landmarks present only in the environment (i.e., IRL). We employed linear mixed-effect models (LME) to compare landmark (d’ score) and route direction recognition across the landmark visualization conditions (i.e., abstract vs. realistic).

Previous and incomplete analyses of the landmark and route knowledge tasks were presented in Kapaj et al. (2022). In this novel contribution, we present several analyses using LME or generalized LME (GLME) on collected data relating to spatial ability and environmental familiarity, the reconstruction of landmark order, landmark distance and direction estimation tasks, and all aspects regarding the role of visual attention.

LME and GLME models were implemented in R (v.4.2.1) using the *lme4* package (Bates et al., 2015). To identify the statistical model that would converge for each dependent variable, we started with a model that includes the dependent variable as a response variable with no fixed effects and a maximal random effect structure imposed by the experimental design (Barr et al., 2013). If the model did not reach convergence, we iteratively reduced the random effect structure by excluding random slopes and intercepts until the model converged. If more than one model converged, we selected the best-fit model according to the Akaike information criterion (AIC). After identifying the random effect structure for each dependent variable and before adding the fixed effects to the selected model, we centered the continuous variables at the mean value and sum-coded dichotomous categorical variables to −0.5 and 0.5. Post-hoc analyses of significant interactions found in LME and GLME models’ were performed using the *emmeans* package (Lenth, 2023). Specifically, we used the *pairs()* method for factor-to-factor interactions to compare the estimated marginal means of one factor with another. We also ran the *emtrends()* function for factor-to-covariate interactions to compare the estimates of the covariate slope trend for each level of the factors. P-values for the models and the post-hoc analyses were calculated using the Satterthwaite approximation for degrees of freedom, as it produces acceptable levels of Type I error (Luke, 2017; Singmann & Kellen, 2019), implemented using the *summary()* function of the *lmerTest* package (Kuznetsova et al., 2017). The model plots were produced using the *ggplot2* package (Wickham, 2016).

## 3. Results

### 3.1. Navigation performance

We examined participants’ navigation accuracy to assess whether navigating with different landmark visualization styles would influence this measure of navigation performance. Task accuracy was measured in terms of participants’ navigation errors, that is, deviations from following the predefined route or failure to identify the task-relevant landmarks. There were only two failures to identify navigation-relevant landmarks, one per visualization condition, committed by two participants. Additionally, there were, in total, eight wrong turns at intersections across five participants. Three of these wrong turns were taken when navigating with landmarks depicted as abstract 3D buildings and five wrong turns when navigating with realistic 3D buildings. As there were only a few navigation errors out of the 460 total navigated intersections (10 per 46 subjects), we did not perform any statistical analysis on these. Navigation performance was thus very high in both conditions.

As part of navigation performance, we also analyzed participants’ completion time – the total time needed to complete the navigation task with each landmark visualization condition. The total navigation task completion time across conditions ranged from 10.2 to 15.1 minutes (*M* = 12.5, *SD* = 1.17). A paired t-test revealed that participants’ completion time was not significantly different (*t(45)* = −0.09, *p* = .9) when navigating with the realistic (*M* = 6.27, *SD* = 1.55) compared to abstract (*M* = 6.23, *SD* = 1.44) landmark visualization style. To account for route segments across visualization styles having different lengths, which might have impacted obtained results, we calculated route segment completion time as a percentage of total navigation completion time ([segment time/total time] * 100). A paired t-test assessing route segment completion times revealed no significant results either (*t(45)* = −0.05, *p* = 1) between conditions (realistic: *M* = 50.1, *SD* = 11.1; abstract: *M* = 49.9, *SD* = 11.1); indicating that the counterbalancing of the conditions took care of possible differences in completion time that might have otherwise arisen due to different segment lengths.

### 3.2. Visual attention

#### Area of interest (AOI): environment

We analyzed participants’ visual attention in the environment using normalized fixation duration (nFD) in the AOI as a response variable (i.e., the percentage of time spent fixating on each AOI over the course of the navigated route) and the experimental condition, including spatial abilities (by QSS score), and the familiarity with the study area as fixed effects. We also included two-way interactions of the experimental condition with the QSS score and familiarity. The selected model included a by-subject random intercept.

Contrary to our first hypothesis (H1), the model results (Table 1) showed that landmark visualization style did not influence wayfinders’ distribution of visual attention on the environment (*β* = −0.85, *SE* = 0.82, *t* = −1.04, *p* = .303). Furthermore, the model revealed that the QSS score (*β* = 1.51, *SE* = 0.87, *t* = 1.73, *p* = .090) and familiarity (*β* = 0.98, *SE* = 1.44, *t* = 0.68, *p* = .501) were not significant predictors of nFD in the environment. However, the model results revealed a significant interaction between the landmark visualization style and the QSS score (*β* = 1.10, *SE* = 0.49, *t* = 2.23, *p* = .031) and between the landmark visualization style and environmental familiarity (*β* = −1.72, *SE* = 0.82, *t* = −2.10, *p* = .041).

**Table 1.** Normalized fixation duration (nFD) in the environment AOI is predicted by the interactions of QSS score and familiarity with the visualization conditions.

nFD in the environment AOI [%]				
*Predictors*	*Estimate*	*Std. Error*	*t-value*	*p-value*
(Intercept)	39.20	1.44	27.17	**<.001**
Condition [Realistic]	−0.85	0.82	−1.04	.303
QSS score	1.51	0.87	1.73	.090
Familiarity [Familiar]	0.98	1.44	0.68	.501
Condition [Realistic] : QSS score	1.10	0.49	2.23	.**031**
Condition [Realistic] : Familiarity [Familiar]	−1.72	0.82	−2.10	.**041**

Significant p-values (|p| < .05) in **bold**.

**Figure 3.**
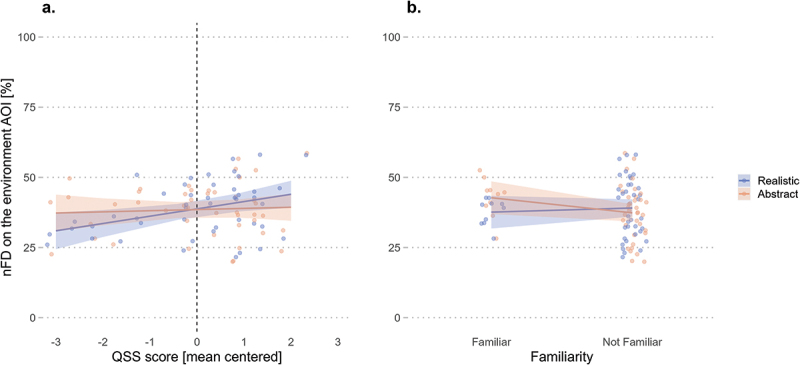
The normalized fixation duration (nFD) in the environment increases with higher spatial abilities when wayfinders navigate with realistic 3D landmarks (a). However, this is not influenced by environmental familiarity across conditions (b). Shaded areas correspond to 95% confidence intervals (CIs).

Post-hoc analyses revealed a significant influence of the QSS slope trend estimates on the environment nFD for the realistic landmarks (*emtrend* = 2.60, *SE* = 0.10, *t* = 2.61, *p* = .011), but not for the abstract landmarks (*emtrend* = 0.41, *SE* = 0.10, *t* = 0.41, *p* = .681). The contrast between the two slopes revealed a significant difference (*β* = 2.19, *SE* = 0.98, *t* = 2.23, *p* = .031), showing that the realistic condition has a marginal advantage of 2.19% in fixation duration in the environment per unit increase in spatial abilities relative to the abstract condition. In other words, these results indicate that in parallel to wayfinders’ higher spatial abilities, their visual attention to the environment increases when navigating with realistic but not abstract 3D landmarks (Figure 3a). These post-hoc interaction analyses also revealed that familiarity was not a predictor of visual attention to the environment, as the differences in estimated marginal means between the realistic and abstract conditions were neither significant for the familiar wayfinders (*emmeans* = −5.13, *SE* = 2.88, *t* = −1.78, *p* = .082) nor those unfamiliar with the traversed environment (*emmeans* = 1.73, *SE* = 1.52, *t* = 1.14, *p* = .262). As the individual slope trends were not significant, the interaction effect in the model is explained by the slightly opposite slope directions of familiarity in each condition (Figure 3b).

#### 3.2.1. Areas of interest (AOI): mobile map

We analyzed participants’ visual attention on the mobile map using normalized fixation durations (nFD) on the AOI as a response variable and the experimental condition as the main effect. We also included the QSS score and environmental familiarity as fixed effects and two-way interaction terms between them and the experimental condition. The model included a by-subject random intercept.

Contrary to our hypothesis (H1), the model results did not reveal any significant influence of the landmark visualization style on the nFD on the mobile map display (*β* = −0.20, *SE* = 0.53, *t* = −0.38, *p* = .704). Additionally, the QSS score, familiarity, and their interactions with the condition were not significant predictors of the nFD on the map AOI (Table 2).

**Table 2.** Normalized fixation duration (nFD) on the mobile map AOI is not predicted by the fixed effects or the interactions between the fixed effects.

nFD on the mobile map AOI [%]				
*Predictors*	*Estimate*	*Std. Error*	*t-value*	*p-value*
(Intercept)	23.06	1.23	18.73	**<.001**
Condition [Realistic]	−0.20	0.53	−0.38	.704
QSS score	−1.39	0.74	−1.88	.067
Familiarity [Familiar]	−0.63	1.23	−0.52	.609
Condition [Realistic] : QSS score	−0.55	0.32	−1.71	.095
Condition [Realistic] : Familiarity [Familiar]	0.29	0.53	0.55	.583

Significant p-values (|p| < .05) in **bold**.

#### 3.2.2. Area of interest (AOI): landmark

We analyzed participants’ visual attention to the landmarks by using normalized fixation durations (nFD) on the AOI as a response variable and the experimental condition, QSS score, and environmental familiarity as fixed effects. Also, the model included two-way interaction terms of condition with the QSS score and familiarity and a by-subject random intercept.

**Figure 4.**
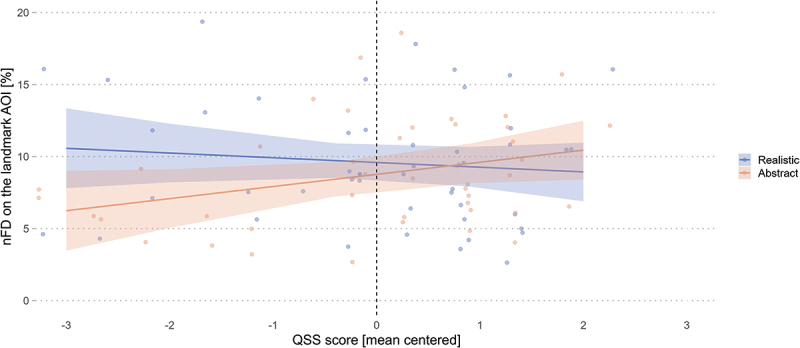
Realistic landmarks prompt longer normalized fixation duration (nFD) on the landmark AOI for low-spatial-ability wayfinders. Shaded areas correspond to 95% CIs.

The model results did not reveal a significant influence of the experimental condition on the nFD on the landmarks, opposing our first hypothesis. In addition, the model results revealed that the QSS score, the familiarity with the study area, and the interaction of familiarity with the condition were not significant predictors either (Table 3). However, there was a borderline interaction effect between the QSS score and condition (*β* = −0.58, *SE* = 0.30, *t* = −1.96, *p* = .054). Post-hoc analyses of this interaction revealed a marginally significant influence of the QSS slope trend estimates for the abstract (*emtrend* = 0.84, *SE* = 0.42, *t* = 1.99, *p* = .050) but not for the realistic (*emtrend* = −0.33, *SE* = 0.42, *t* = −0.78, *p* = .439) 3D landmark visualization conditions. While a unit increase in spatial abilities suggests a decrease in visual attention on the landmarks AOI for the realistic condition – with a marginal difference of −1.17% relative to the abstract condition – the contrast between the condition slopes did not differ significantly (*β* = −1.17, *SE* = 0.60, *t* = −1.96, *p* = .054). Further examination of Figure 4 to better understand this results pattern reveals that navigators with low spatial abilities have lower landmark fixation durations when these landmarks are presented in an abstract manner. The realistic landmark visualization style increased landmark fixation duration for participants with low spatial abilities, bringing them closer to the navigators with higher spatial ability, who were unaffected by the landmark visualization style. Overall, the results showed that the realistic condition prompts longer visual attention on the landmark AOI for wayfinders with low spatial abilities but not for those with higher spatial abilities.

**Table 3.** Normalized fixation duration (nFD) on the landmark AOI is not predicted by condition, QSS score, familiarity, or fixed-effect interactions.

nFD on the landmarks AOI [%]				
*Predictors*	*Estimate*	*Std. Error*	*t-value*	*p-value*
(Intercept)	9.50	0.50	19.17	**<.001**
Condition [Realistic]	0.71	0.50	1.44	.154
QSS score	0.26	0.30	0.86	.393
Familiarity [Familiar]	0.58	0.50	1.16	.248
Condition [Realistic] : QSS score	−0.58	0.30	−1.96	.054
Condition [Realistic] : Familiarity [Familiar]	0.53	0.50	1.07	.287

Significant p-values (|p| < .05) in **bold**.

### 3.3. Spatial learning

#### 3.3.1. Landmark knowledge

**Recognition of landmarks seen in the environment** was analyzed using d’ as a response variable and the fixed effects of experimental condition, QSS score, normalized fixation duration (nFD) on each of the three AOIs, and environmental familiarity. We also included two-way interaction terms of the condition with the nFD on each AOI. Furthermore, we added two-way interaction terms of the condition with the QSS score and environmental familiarity and two-way interactions of the QSS score with familiarity and nFD on each AOI. The selected model included a by-subject random intercept.

Contrary to our first hypothesis, the model results (Table 4) did not reveal a significant relationship between the visualization condition and the recognition of landmarks seen in the environment (*β* = 0.05, *SE* = 0.08, *t* = 0.71, *p* = .482). The model revealed a significant main effect of environmental familiarity (*β* = 0.17, *SE* = 0.08, *t* = 2.22, *p* = .029), indicating that wayfinders indeed have a higher recognition accuracy of landmarks seen in the environment that is familiar to them. Furthermore, and in line with our second hypothesis, we found that experimental condition interacted with both nFD on the environment AOI (*β* = 0.03, *SE* = 0.01, *t* = 2.06, *p* = .043) and nFD on the landmark AOI (*β* = 0.05, *SE* = 0.02, *t* = 2.50, *p* = .014).

**Table 4.** Recognition of environment landmarks is predicted by environmental familiarity and the interactions between nFD in the environment and landmark AOIs with the condition.

Recognition of environment landmarks [d’]				
*Predictors*	*Estimate*	*Std. Error*	*t-value*	*p-value*
(Intercept)	1.32	0.08	17.38	**<.001**
Condition [Realistic]	0.05	0.08	0.71	.482
QSS score	0.06	0.07	0.82	.417
Familiarity [Familiar]	0.17	0.08	2.22	.**029**
nFD environment	<-0.01	0.01	−0.02	.981
nFD mobile map	−0.01	0.02	−0.58	.565
nFD landmark	0.04	0.02	1.63	.108
Condition [Realistic] : QSS score	−0.06	0.05	−1.17	.245
Condition [Realistic] : Familiarity [Familiar]	0.01	0.08	0.12	.905
Condition [Realistic] : nFD environment	0.03	0.01	2.06	.**043**
Condition [Realistic] : nFD mobile map	0.02	0.02	1.28	.206
Condition [Realistic] : nFD landmark	0.05	0.02	2.50	.**014**
QSS score : Familiarity [Familiar]	−0.04	0.07	−0.58	.566
QSS score : nFD environment	<-0.01	0.01	−0.11	.913
QSS score : nFD mobile map	0.02	0.01	1.42	.161
QSS score : nFD landmark	0.03	0.02	1.70	.093

nFD = normalized fixation duration [%]; Significant p-values (|p| < .05) in **bold**.

**Figure 5.**
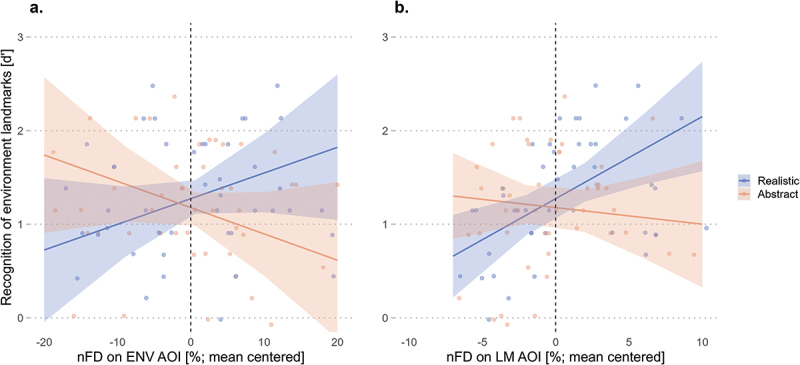
Longer normalized fixation duration (nFD) in the environment (a) and on the landmark (b) is associated with improved recognition accuracy of landmarks seen in the environment when wayfinders navigate with realistic 3D landmarks. Shaded areas correspond to 95% CIs.

Post-hoc analyses were conducted to investigate the influence of slope trend estimates for the nFD on the environment and the nFD on the landmarks on recognizing environment landmarks across conditions. While the results revealed that the nFD on the environment was not a significant predictor across the realistic (*emtrend* = 0.03, *SE* = 0.02, *t* = 1.45, *p* = .150) and the abstract (*emtrend* = −0.03, *SE* = 0.02, *t* = −1.37, *p* = .174) visualization styles, the contrast between the two slopes revealed a significant difference (*β* = 0.06, *SE* = 0.03, *t* = 2.06, *p* = .043). This difference in contrast slopes shows that the realistic condition has a marginal advantage of 0.06 in d’, relative to the abstract condition, for recognizing landmarks seen in the environment per percentage increase of visual attention in the environment (Figure 5a). Similarly, the post-hoc analysis results revealed that the nFD on the landmarks significantly predicts the recognition of landmarks seen in the environment for the realistic landmark (*emtrend* = 0.09, *SE* = 0.03, *t* = 3.09, *p* = .003) but not for the abstract landmark (*emtrend* = −0.02, *SE* = 0.03, *t* = −0.56, *p* = .579) conditions. Additionally, the contrast between the two slopes revealed a significant difference (*β* = 0.11, *SE* = 0.04, *t* = 2.50, *p* = .015), showing that the realistic condition has a marginal advantage of 0.11 in d,’ relative to the abstract condition, for recognizing landmarks seen in the environment per percentage increase of visual attention on the landmarks (Figure 5b).

**Recognition of landmarks seen on the mobile map** was analyzed using a model with d’ values as a response variable and the fixed effects of experimental condition, QSS score, normalized fixation duration (nFD) on each AOI, and environmental familiarity with the study area. Furthermore, we included three interactions between the condition with the nFD on each AOI, interactions of the condition with QSS score and familiarity, and interactions of QSS with familiarity and nFD on each AOI. The selected model included a by-subject random intercept.

Contrary to our first hypothesis, the model results did not reveal a significant influence of experimental conditions on wayfinders’ landmark recognition when seen on the mobile map (*β* = 0.13, *SE* = 0.08, *t* = 1.64, *p* = .109). Furthermore, the model revealed that participants’ visual attention on each AOI was also not a significant predictor of the recognition performance of landmarks on the mobile map (Table 5).

**Table 5.** The recognition accuracy of landmarks seen on the mobile map is predicted by the QSS score and its interaction with nFD on the environment and on the mobile map.

Recognition of mobile map landmarks [d’]				
*Predictors*	*Estimate*	*Std. Error*	*t-value*	*p-value*
(Intercept)	1.73	0.10	16.71	**<.001**
Condition [Realistic]	0.13	0.08	1.64	.109
QSS score	0.29	0.10	2.94	.**005**
Familiarity [Familiar]	0.14	0.10	1.32	.193
nFD environment	<-0.01	0.02	−0.23	.822
nFD mobile map	0.01	0.02	0.42	.674
nFD landmark	0.01	0.03	0.21	.838
Condition [Realistic] : QSS score	−0.02	0.05	−0.36	.719
Condition [Realistic] : Familiarity [Familiar]	−0.02	0.08	−0.19	.848
Condition [Realistic] : nFD environment	−0.02	0.02	−1.44	.155
Condition [Realistic] : nFD mobile map	−0.01	0.02	−0.77	.448
Condition [Realistic] : nFD landmark	0.02	0.02	0.66	.509
QSS score : Familiarity [Familiar]	0.06	0.10	0.64	.524
QSS score : nFD environment	0.03	0.01	2.89	.**005**
QSS score : nFD mobile map	0.03	0.01	2.49	.**015**
QSS score : nFD landmark	0.03	0.02	1.43	.157

nFD = normalized fixation duration [%]; Significant p-values (|p| < .05) in **bold**.

**Figure 6.**
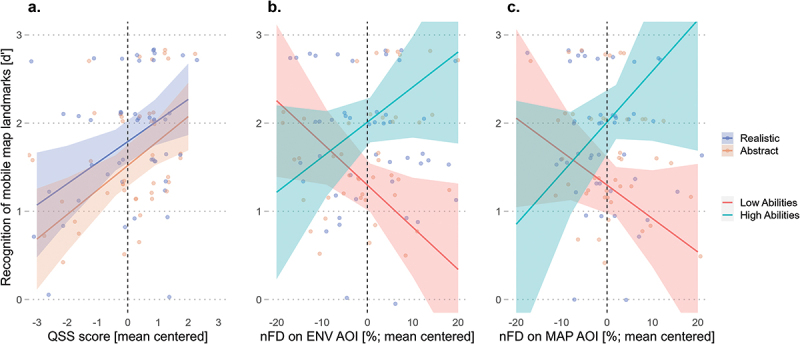
Improved recognition accuracy performance of landmarks featured on the mobile map with increased QSS score, regardless of the landmark visualization style (a); improved performance for high-spatial-ability wayfinders and deteriorated performance for low-spatial-ability wayfinders with increased attention in the environment (b) and on the mobile map (c) AOIs. Shaded areas correspond to 95% CI.

The results also revealed that the QSS score significantly predicted performance (*β* = 0.29, *SE* = 0.10, *t* = 2.94, *p* = .005) but did not interact with the experimental conditions (*β* = −0.02, *SE* = 0.05, *t* = −0.36, *p* = .719). The effect of the QSS score indicates that wayfinders’ recognition accuracy of landmarks depicted on the mobile map improved with increased spatial abilities, regardless of the landmark visualization style (Figure 6a). Additionally, the results revealed significant interaction effects between QSS score and nFD on the environment (*β* = 0.03, *SE* = 0.01, *t* = 2.89, *p* = .005) and on the mobile map (*β* = 0.03, *SE* = 0.01, *t* = 2.49, *p* = .015). Indicating that while, on the one hand, the recognition performance of landmarks featured on the mobile map improves when high-spatial-ability wayfinders look longer at the environment (Figure 6b) and the mobile map (Figure 6c) AOIs, on the other hand, the performance deteriorates when low-spatial-ability wayfinders focus longer on these AOIs.

#### 3.3.2. Route knowledge

**Route knowledge** was assessed with a GLME model to analyze the recall accuracy of route directions. The GLME model includes route direction accuracy as the response variable (binomial, correct vs. incorrect) and the experimental condition, nFD on each AOI, QSS score, and participants’ familiarity with the study area as fixed effects. In addition, we included two-way interaction terms between the experimental condition and the nFD on the AOIs, the QSS score, and the environmental familiarity, and two-way interactions between the QSS score and environmental familiarity and the nFD on each AOI. The model included a by-subject random intercept and slope for the condition.

**Figure 7.**
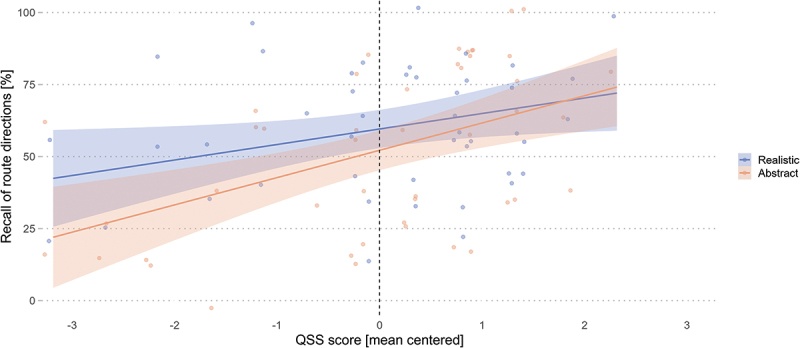
Route direction recall accuracy improves with increased spatial abilities, regardless of the landmark visualization style. Shaded areas correspond to 95% CIs.

Contrary to our first hypothesis that participants would have better route recall accuracy with realistic 3D landmarks, the model result revealed no significant effects of the visualization condition (*β* = 0.16, *SE* = 0.13, *z* = 1.23, *p* = .219). Similarly, in contrast to our second hypothesis, the model results revealed no significant main effect of the nFD on each AOI nor a significant interaction with the experimental condition (Table 6). However, the results revealed that the QSS score (*β* = 0.38, *SE* = 0.13, *z* = 2.99, *p* = .003) and familiarity (*β* = 0.29, *SE* = 0.13, *z* = 2.22, *p* = .027) were significant predictors but did not interact with the landmark visualization style – even though Figure 7 shows a numerical improvement in route direction recall when low-spatial-ability wayfinders navigated with the realistic landmarks.

**Table 6.** Route direction recall accuracy is predicted by the QSS score and familiarity.

Route direction recall accuracy				
*Predictors*	*Estimate*	*Std. Error*	*z-value*	*p-value*
(Intercept)	0.38	0.13	2.93	.**003**
Condition [Realistic]	0.16	0.13	1.23	.219
QSS score	0.38	0.13	2.99	.**003**
Familiarity [Familiar]	0.29	0.13	2.22	.**027**
nFD environment	−0.04	0.02	−1.80	.072
nFD mobile map	−0.04	0.03	−1.20	.230
nFD landmark	−0.05	0.04	−1.28	.199
Condition [Realistic] : QSS score	−0.10	0.09	−1.19	.233
Condition [Realistic] : Familiarity [Familiar]	−0.05	0.13	−0.42	.678
Condition [Realistic] : nFD environment	−0.01	0.02	−0.49	.624
Condition [Realistic] : nFD mobile map	<-0.01	0.03	−0.01	.992
Condition [Realistic] : nFD landmark	0.04	0.04	1.20	.229
QSS score : Familiarity [Familiar]	−0.03	0.13	−0.26	.798
QSS score : nFD environment	0.02	0.02	1.18	.238
QSS score : nFD mobile map	0.02	0.02	0.92	.356
QSS score : nFD landmark	0.03	0.03	1.13	.257

nFD = normalized fixation duration [%]; Significant p-values (|p| < .05) in **bold**.

**The reconstruction of the landmark sequence** was analyzed using a generalized linear mixed effect model (GLME) with the reconstruction of the order of seen landmarks as the response variable (binomial, correct vs. incorrect) and the experimental condition, QSS score, and environmental familiarity as fixed effects. In addition, the GLME model included two-way interaction terms of the condition with the QSS score and familiarity.^1^^1^The fixed effects of nFD on each AOI and their two-way interactions with the condition and the QSS score were removed from the final model due to convergence issues. The model included a by-subject random intercept and slope for the condition.

Contrary to our first hypothesis, the GLME model revealed no significant relationship between the visualization condition and the recognition of landmarks sequence as seen during the wayfinding task (*β* = 0.17, *SE* = 0.25, *z* = 0.68, *p* = .498; Table 7). However, the model results revealed a significant main effect of environmental familiarity but no interaction with the experimental conditions. Additionally, the results revealed that the QSS score was a significant predictor (*β* = 0.41, *SE* = 0.16, *z* = 2.66, *p* = .008), while the interaction with the landmark visualization condition approached significance (*β* = −0.28, *SE* = 0.14, *z* = −1.94, *p* = .052) for the reconstruction of the landmarks sequence.

**Table 7.** Reconstruction of landmarks sequence is predicted by QSS and familiarity.

Reconstruction of landmarks’ sequence				
*Predictors*	*Estimate*	*Std. Error*	*z-value*	*p-value*
(Intercept)	0.17	0.25	0.68	.498
Condition [Realistic]	0.12	0.23	0.50	.619
QSS score	0.41	0.16	2.66	.**008**
Familiarity [Familiar]	0.54	0.26	2.21	.**034**
Condition [Realistic] : QSS score	−0.28	0.14	−1.94	.052
Condition [Realistic] : Familiarity [Familiar]	−0.15	0.24	−0.65	.515

Significant p-values (|p| < .05) in **bold**.

**Figure 8.**
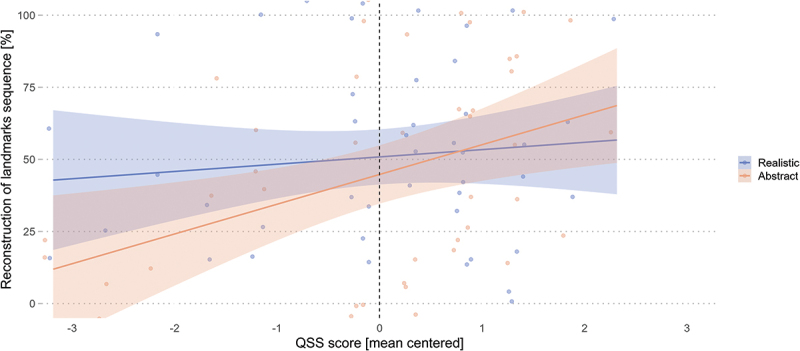
Landmark sequence reconstruction improves with increased spatial abilities regardless of the landmark visualization styles and improves when low-spatial-ability wayfinders navigate with realistic landmarks. Shaded areas correspond to 95% CIs.

To follow up on this possible interaction effect, post-hoc analyses were conducted to investigate the influence of QSS slope trend estimates on sequence knowledge across conditions. These results revealed that a QSS increase was associated with a significant improvement in landmark sequence knowledge for the abstract landmark condition (*emtrend* = 0.70, *SE* = 0.24, *z* = 2.84, *p* = .004) but not for the realistic landmark condition (*emtrend* = 0.13, *SE* = 0.17, *z* = 0.76, *p* = .446). While a unit increase in spatial abilities suggests a decrease in the recognition accuracy of landmark sequence for the realistic condition, with a marginal difference of −0.56 relative to the abstract condition, the contrast between the condition slopes did not differ significantly (*β* = −0.56, *SE* = 0.29, *z* = −1.94, *p* = .052). The close-to-significant interaction in the main model, the post-hoc analyses on QSS slope trend estimates, and the visual inspection of Figure 8 show that realistic landmark visualization is most helpful in landmark sequence memory for participants with low spatial abilities, and this difference between the conditions diminishes with increased spatial abilities.

#### 3.3.3. Survey knowledge

**Direction estimation** among the REL landmarks was analyzed using an LME with the direction estimation error expressed in angular degrees as the response variable and the experimental condition, nFD on each AOI, QSS score, and environmental familiarity as fixed effects. We further included the perspective-taking and spatial orientation test (PTSOT) error as a fixed effect to control for the individual survey learning ability as a baseline, thought to underline participants’ direction estimation performance. We excluded one participant whose PTSOT error was higher than the 90-degree chance performance. In addition to the main effects, the model included two-way interactions between the condition and the QSS score, PTSOT error, familiarity, and the nFD in each AOI, and other two-way interactions between the QSS score and the familiarity and with the nFD on each AOI. The selected model included a by-subject random intercept and slope for the condition.

Contrary to our first hypothesis, the model results (Table 8) did not reveal a significant influence of landmark visualization style on direction estimation among landmarks (*β* = −3.20, *SE* = 2.14, *t* = −1.50, *p* = .143). Furthermore, in contrast to our second hypothesis, the model results revealed that the nFD on each AOIs was not a significant predictor of wayfinders’ direction estimation accuracy. However, the model results revealed a significant interaction between the landmark visualization style and participants’ QSS score (*β* = 5.21, *SE* = 1.52, *t* = 3.42, *p* = .001) but not a significant main effect of the QSS score (*β* = −2.08, *SE* = 3.31, *t* = −0.63, *p* = .532).

**Table 8.** Direction estimation accuracy is influenced by the interaction between landmark visualization conditions and the QSS score.

	Direction estimation error [angular degree]			
*Predictors*	*Estimate*	*Std. Error*	*t-value*	*p-value*
(Intercept)	42.21	3.49	12.09	**<.001**
Condition [Realistic]	−3.20	2.14	−1.50	.143
QSS score	−2.08	3.31	−0.63	.532
PTSOT error	0.19	0.27	0.71	.482
Familiarity [Familiar]	−6.58	3.47	−1.90	.065
nFD environment	−0.96	0.58	−1.67	.100
nFD mobile map	−0.75	0.73	−1.02	.312
nFD landmark	−0.01	0.82	−0.01	.989
Condition [Realistic] : QSS score	5.21	1.52	3.42	.**001**
Condition [Realistic] : PTSOT error	0.18	0.16	1.09	.280
Condition [Realistic] : Familiarity [Familiar]	−0.73	2.22	−0.33	.744
Condition [Realistic] : nFD environment	0.16	0.42	0.39	.699
Condition [Realistic] : nFD mobile map	0.29	0.55	0.53	.601
Condition [Realistic] : nFD landmark	−0.65	0.73	−0.89	.377
QSS score : Familiarity [Familiar]	0.12	3.29	0.04	.972
QSS score : nFD environment	0.25	0.36	0.71	.483
QSS score : nFD mobile map	0.10	0.43	0.23	.816
QSS score : nFD landmark	−0.57	0.53	−1.09	.282

nFD = normalized fixation duration [%]; Significant p-values (|p| < .05) in **bold**.

**Figure 9.**
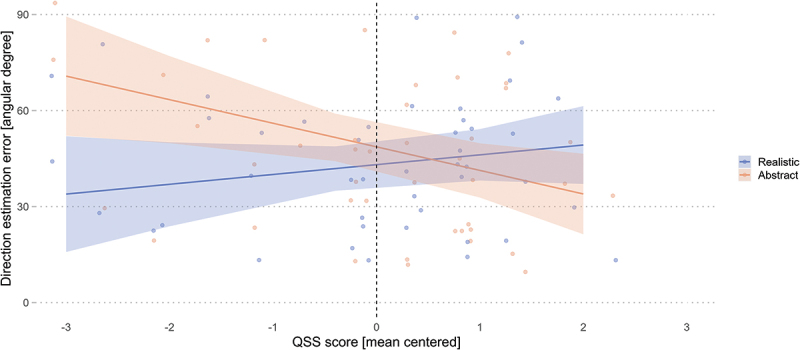
Direction estimation error improves when wayfinders with low spatial abilities navigate with realistic landmarks. Shaded areas correspond to 95% CI.

Post-hoc analyses on the interaction effect revealed no influence of the QSS slope trend estimates on the direction estimation error for the realistic landmark condition (*emtrend* = 3.13, *SE* = 3.64, *t* = 0.86, *p* = .395), and a marginally significant influence for the abstract landmark condition (*emtrend* = −7.30, *SE* = 3.64, *t* = −2.00, *p* = .050). The contrast between the two slopes revealed a significant difference (*β* = 10.4, *SE* = 3.05, *t* = 3.42, *p* = .002), showing that the abstract condition has a marginal decrease of 10.4° in the direction estimation error per unit increase in spatial abilities relative to the realistic condition. Taken together, the significant difference in slope contrasts and the visual examination of Figure 9 reveals that the realistic landmark visualization style improves the direction estimation error for wayfinders with low spatial abilities, and this difference between the conditions diminishes for participants with high spatial abilities.

**Distance estimation** among REL landmarks was analyzed using an LME with the distance estimation error expressed in meters as the response variable and the experimental condition, nFD on each AOI, QSS score, and environmental familiarity as fixed effects. We added two-way interaction terms between the condition and the QSS score, familiarity, and the nFD on each AOI, and two-way interactions of the QSS score and the environmental familiarity and with the nFD on the three AOIs. The selected model included a by-subject random intercept and slope for the condition.

**Figure 10.**
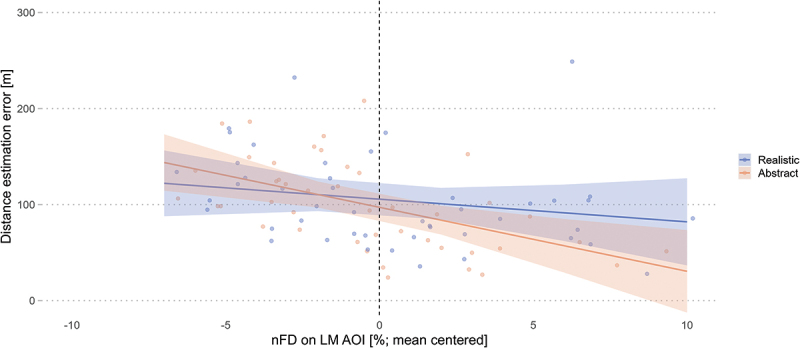
Longer visual attention on the landmarks AOI improves wayfinders’ distance estimation error, regardless of the landmark visualization style. Shaded areas correspond to 95% CI.

Contrary to our first hypotheses, the model revealed non-significant results for the influence of landmark visualization style (*β* = 6.91, *SE* = 4.55, *t* = 1.52, *p* = .136) on participants’ distance estimation error (Table 9). In line with our second hypothesis, we did find a significant main effect of the normalized fixation duration (nFD) on the landmark AOI (*β* = −4.51, *SE* = 1.59, *t* = −2.84, *p* = .006), but no interactions with the experimental conditions (*β* = 2.15, *SE* = 1.42, *t* = 1.51, *p* = .136; Figure 10). These results indicate that increased visual attention on task-relevant landmarks in the environment improves wayfinders’s knowledge about the distance between these landmarks, regardless of the landmark visualization style depicted on the mobile map.

**Table 9.** The distance estimation error is predicted by the nFD on the landmark AOI.

	Direction estimation error [angular degree]			
*Predictors*	*Estimate*	*Std. Error*	*t-value*	*p-value*
(Intercept)	105.76	6.73	15.71	**<.001**
Condition [Realistic]	6.91	4.55	1.52	.136
QSS score	−3.15	6.45	−0.49	.627
Familiarity [Familiar]	7.72	6.68	1.16	.255
nFD environment	1.31	1.10	1.20	.235
nFD mobile map	1.35	1.46	0.93	.357
nFD landmark	−4.51	1.59	−2.84	.**006**
Condition [Realistic] : QSS score	−0.33	3.12	−0.10	.917
Condition [Realistic] : Familiarity [Familiar]	4.73	4.65	1.02	.315
Condition [Realistic] : nFD environment	−0.21	0.86	−0.24	.808
Condition [Realistic] : nFD mobile map	0.73	1.13	0.65	.520
Condition [Realistic] : nFD landmark	2.15	1.42	1.51	.136
QSS score : Familiarity [Familiar]	−6.81	6.24	−1.09	.281
QSS score : nFD environment	0.76	0.70	1.09	.281
QSS score : nFD mobile map	1.11	0.87	1.27	.207
QSS score : nFD landmark	0.92	1.11	0.83	.409

nFD = normalized fixation duration [%]; Significant p-values (|p| < .05) in **bold**.

## 4. Discussion

The current study presents the results of a real-world navigation experiment conducted to examine the influence of landmark visualization style on mobile maps, comparing realistic or abstract 3D landmark buildings on wayfinders’ navigation task performance, visual attention, and spatial learning. We find mixed support for our hypotheses, with some effects on spatial learning seen in people with lower spatial abilities and some modulation of visual attention, but no differences in navigation performance between landmark visualization style conditions.

### 4.1. Navigation performance

Contrary to our hypothesis that wayfinders will have a better navigation performance when navigating with realistic 3D landmarks, as Liao et al. (2017) and Lokka and Çöltekin (2019) suggest, the results revealed no differences in the number of wrong turns, failure to identify the task-relevant landmarks or navigation completion time across the landmark visualization conditions. In general, the navigation performance was at ceiling level, and thus, the lack of differences in navigation performance is likely because both the abstract and realistic 3D landmark visualization provided the necessary visual information to guide novice wayfinders in a mostly unfamiliar urban environment (Ramanoël et al., 2022). After the navigation task, wayfinders stated their preference for the landmark visualization style, with 82.6% favoring the realistic depiction, 8.7% the abstract depiction, and the remaining 8.7% were neutral. The navigation performance and visualization preference results align with previous research findings that while participants prefer realistic 3D depictions, they do not always perform better with them (Hegarty et al., 2009; Kray et al., 2003; Liao et al., 2017; Oulasvirta et al., 2009; Plesa & Cartwright, 2008). While wayfinders did not benefit from the cartographic design changes on navigation-relevant landmarks shown on their navigation aid in terms of navigation performance, it is reassuring that adding more visual information to the preferred realistic visualization did not adversely affect navigation efficiency, which was a risk highlighted in other studies (Dillemuth, 2005; Liao et al., 2017; Plesa & Cartwright, 2008). Our findings are also consistent with the results of Ramanoël et al. (2022), who did not find any differences in navigation performance between the abstract objects and textured 3D features located at the intersections of a “Y” shaped virtual maze environment. Overall, the high navigation performance in our real-world, mobile map-assisted navigation study and the previous findings that show improved performance when landmarks are present compared to when no landmark information is provided to the wayfinders (Cliburn et al., 2007; Heft, 1979; Jansen-Osmann & Fuchs, 2006; Sharma et al., 2017; Waller & Lippa, 2007) fits with the call for landmarks to feature prominently on mobile maps during map-assisted navigation.

### 4.2. Visual attention

We found limited support for our visual attention hypothesis that the realistic 3D landmark visualization will affect wayfinders’ attention modulation between the mobile map, the task-relevant landmarks, and the environment. Specifically, wayfinders with higher spatial abilities exhibited longer attention to the traversed environment when the mobile map featured realistic 3D landmarks, which means that they must have spent less time attending to the other AOIs. Specifically, participants in the realistic landmark condition show a reduced proportion of fixation time tendency on landmarks and the mobile map, although it is important to note that these changes did not reach significance. This is likely because the extra time spent looking at the environment was taken evenly from all other AOIs, thereby diminishing a potential effect size. Wayfinders with low spatial abilities exhibited the opposite pattern, with shorter visual attention spans directed to the environment and longer attention on the task-relevant landmarks when navigating with realistic landmarks on the mobile map. Therefore, our hypothesis that realistic landmarks will guide higher visual attention on task-relevant landmarks during navigation is only supported for wayfinders with low spatial abilities, as it was not the case for participants with high spatial abilities. This finding aligns with previous work in that visually salient landmarks do attract and modulate viewers’ bottom-up or stimulus-driven visual attention, but clearly in different ways for individuals with different backgrounds, training, abilities, etc (Richter & Winter, 2014; Wenczel et al., 2017; Wolfe & Horowitz, 2017; Yesiltepe et al., 2021). It makes sense that the low spatial ability group requires more assistance in guiding their attention to task-relevant landmarks and that navigators with greater spatial abilities are able to modulate their attention more independently (Hegarty et al., 2006; Ishikawa, 2023; Montello, 1998). Indeed, people with lower spatial abilities rely more on mobile maps – which was also the case in our study, regardless of the landmark visualization style – and thus are more likely to be prone to negative effects on spatial learning, such as passive reliance on navigation aids and thus lesser engagement with the environment (Dahmani & Bohbot, 2020; Ishikawa, 2019; Ruginski et al., 2019). This indeed calls for future mobile map designs that are user-, task-, and navigation context adaptive (Fabrikant, 2023a, 2023b).

### 4.3. Spatial learning

Regarding spatial learning, we hypothesized that wayfinders’ landmark, route, and survey knowledge would be better when navigating with realistic compared to abstract 3D landmarks. Participants showed similar recognition ability for having seen the landmarks in the environment and landmarks depicted on the mobile map across both visualization styles. There was also no effect of condition on route direction recall. As measures of landmark and route knowledge, the foundations of cognitive spatial representations, these results lend weight to previous findings that that wayfinders do not necessarily perform better with increased realism (Hegarty et al., 2009; Liao et al., 2017; Plesa & Cartwright, 2008; Wilkening & Fabrikant, 2011). This argument also extends to distance estimation, however, unlike the other spatial knowledge measures, performance was very low on this task, and thus, it seems that a single route exposure was insufficient to build any distance knowledge. Distance estimation can be measured in terms of traveled path length or straight-line (as-the-crow-flies) Euclidean distance (Epstein et al., 2017). We asked participants to estimate the straight-line distance between landmarks, which is computed from the traveled path distance, and this translation estimation might have added distance errors to participants’ distance estimation performance (Richardson et al., 1999). However, this is not necessarily an indicator of poor overall survey learning, as participants performed rather well on the direction estimation task. While it is important to know how far it takes to traverse an environment – that is, distance knowledge – it is, arguably, more critical to know the direction of the intended destination (Epstein et al., 2017). Our data shows that realistic landmark visualization on the mobile map improved navigators’ knowledge about landmark configurations for navigators with lower spatial abilities, both in terms of route direction recall and direction estimation accuracy between landmarks.

We also observed an interaction that approached significance (*p* = .052) between landmark visualization style and spatial abilities. Post-hoc analyses revealed that the landmark sequence knowledge improved per unit increase in spatial abilities only for the abstract landmarks (*p* = .004). While the results suggest improvement in landmark sequence knowledge when low-spatial-ability wayfinders were guided by realistic landmarks on the mobile map, high-spatial-ability wayfinders were unaffected by the landmark visualization style (Figure 8). However, the difference between the spatial abilities contrast slopes across conditions was only marginal (*p* = .052), possibly due to an unbalanced participant sample between low and high spatial abilities. These results should not be treated as meaningful since both the interaction and the follow-up analyses did not reach significance. However, our data at least highlights sequence knowledge as a feature of spatial learning to be investigated further with regard to future mobile map design and landmark visualization for different user groups. We hypothesize that in a dedicated sample of users with low spatial abilities, landmark sequence knowledge would improve when navigating with realistic 3D landmarks.

Taken together with the longer visual attention on task-relevant landmarks for wayfinders with low spatial abilities, it seems likely that increased cognitive processing of landmark features resulted in better integration of landmark and spatial information. These results indicate that successful navigation is predicted by the ability to attend to relevant landmarks (Chrastil & Warren, 2012; Hamid et al., 2010) and align with previous findings showing that visually salient features improve participants’ spatial memory (Denis et al., 2014; Fine & Minnery, 2009; Keil et al., 2020).

The lack of direct influence of landmark visualization styles on spatial learning measures can be attributed to the fact that depicting landmarks as 3D building models in both conditions on the mobile map could have provided the necessary visual information to facilitate the basic spatial learning required to solve the navigation task (Chrastil & Warren, 2012; Elias & Paelke, 2008; Kapaj et al., 2023; Liao et al., 2017; Willis et al., 2009). On the other hand, deeper spatial learning about landmark configurations that do not necessarily occur on the first exposure to an environment (Ishikawa & Montello, 2006) was improved for participants with lower spatial abilities.

The strong relationship between spatial ability and spatial learning in almost all of our models (i.e., the recognition of landmarks on the mobile map, the reconstruction of landmark sequence, and the recall of route directions) and the interactions with the landmark visualization style condition described above are in line with previous work on individual differences in navigation ability (Hegarty et al., 2006; Newcombe, 2018; Newcombe et al., 2022). This highlights to mobile map designers the importance of tailored design choices for the target user population, especially in view of our previous work with expert navigators who did not benefit from visualization changes at all (Kapaj et al., 2023), just like the participants with higher spatial ability in the present navigation study. Our results contradict previous findings that added realism hurts performance, especially for users with low spatial abilities (Hegarty et al., 2009; Wilkening & Fabrikant, 2011). On the other hand, our results are in line with the findings of previous studies that the visualization style does not hinder wayfinders with high-spatial abilities (Hegarty et al., 2009; Kapaj et al., 2021, 2023; Lanini-Maggi et al., 2021; Thrash et al., 2019; Wilkening & Fabrikant, 2011).

### 4.4. The influence of visual attention on spatial learning

Regarding the influence of visual attention on spatial learning, we hypothesized that longer visual attention on task-relevant landmarks and environment information would improve users’ spatial learning, whilst longer attention to the mobile map display would reduce acquisition of spatial knowledge (Chrastil & Warren, 2012; Gardony et al., 2013, 2015; Hejtmánek et al., 2018; Kapaj et al., 2023; Koletsis et al., 2017; Willis et al., 2009). We found partial support regarding the influence of visual attention on wayfinders’ spatial learning across the landmark visualization styles. Our results revealed only one main effect of visual attention on distance estimation error across all spatial learning tasks. There were also interaction effects between visual attention and landmark visualization styles for recognizing landmarks in the environment, and interactions with wayfinders’ spatial abilities for recognizing landmarks depicted on the mobile map.

Estimation of the distance between landmarks was improved with increased attention to landmark features in the environment, regardless of the landmark visualization style. Montello (1997) stated that encoding of distance information is contributed to by sensorimotor integration of visual landmark encoding and idiothetic body-based cues about active movement. Hence, increased attention to landmarks facilitated wayfinders’ ability to perceive and represent metric information whilst moving between landmarks during navigation (Montello, 1997). Further, we found that higher visual attention on the environment and landmark AOIs was associated with a better recognition accuracy of the landmarks seen in the environment when wayfinders navigated with the mobile map aid showing realistic 3D landmarks. This result aligns with previous studies, showing that guiding attention to the environment and task-relevant landmarks strengthens the encoding of these features and supports spatial memory formations (Gardony et al., 2013, 2015; Hejtmánek et al., 2018; Willis et al., 2009). It also emphasizes the role of visual saliency – like the case of the realistic landmarks in our study – in improving wayfinders’ recall performance (Fine & Minnery, 2009). Given that increased visual attention to the environment and task-relevant landmarks was the goal, these results taken together suggest that realistic landmarks shown on mobile maps are a promising step toward further strengthening wayfinders’ knowledge about the configuration of the traversed space during map-assisted navigation, which is a feature of more advanced cognitive map-like representations (Chrastil, 2013).

The results for the recognition accuracy of landmarks on the mobile map revealed that wayfinders with high spatial ability exhibited a performance improvement when they looked more at the environment and at the mobile map. The improved performance when high spatial ability wayfinders attend longer to the mobile map indicates that they can efficiently extract information about landmarks from the map, considering their overall reduced time spent attending to the mobile map. In contrast and paradoxically, longer time spent attending to the map for wayfinders with low spatial abilities was associated with worse recall of map-featured landmarks. Hejtmánek et al. (2018) suggested that low-spatial ability wayfinders focus longer on a mobile map during navigation because they are not confident in their spatial abilities, which coincided with reduced navigation performance in their study. Our findings support this explanation by showing that longer map fixation times in low spatial ability participants was also associated with worse landmark recall. One possible reason why the longer fixation time did not facilitate recall is that individuals with poor spatial abilities struggle to extract task-relevant information in a sustained manner.

Our results further revealed that when wayfinders with low spatial abilities exhibited increased attention to the environment, performance in recognizing landmarks that were featured on the mobile map was worse. This result makes sense as the information to solve this task could only be found by either attending to the mobile map or to the landmarks themselves. This finding gives context to our results discussed above that the attention of low spatial ability navigators was directed away from the environment and toward the landmarks overall, showing that this direction of attention results in functional learning changes. Put simply, looking at irrelevant information is associated with poorer spatial knowledge development, whilst engagement with relevant information is better for spatial learning (Chrastil & Warren, 2012; Gardony et al., 2013, 2015; Hejtmánek et al., 2018; Willis et al., 2009).

### 4.5. Limitations and future directions

Some limitations of the present real-world route-following navigation study should be pointed out and considered by future similar research. First, we focused only on two depiction styles of local landmarks placed at intersections where a navigation decision was required. The lack of direct differences found in visual attention and spatial learning across the implemented landmark visualization styles could be explained by visual differences between them being too subtle and by the fact that they both provide the necessary visuospatial cues for successful navigation (Ramanoël et al., 2022). Therefore, future work could consider other and more distinctly different cartographic visualization styles (Elias & Paelke, 2008) for saliently depicting local and global landmarks in mobile maps, whose distinction is considered equally important (Credé et al., 2020; Steck & Mallot, 2000; Yesiltepe et al., 2021) to assess wayfinders’ spatial learning.

Second, our study investigated the influence of two landmark visualization styles on wayfinders’ intentional learning. Even though we did not reveal any specific information on the utilized tests, the fact that users were aware that their knowledge would be tested might have motivated them to pay more attention and learn about the environmental configuration regardless of the landmark visualization style. Previous research has shown that when wayfinders are informed that their environmental knowledge will be tested, they exhibit different spatial learning strategies and visual attention behavior as opposed to when they are not made aware of upcoming assessments (Chrastil & Warren, 2012; Van Asselen et al., 2006; Wenczel et al., 2017). Therefore, future research should consider investigating the influence of landmark visualization style on wayfinders’ incidental spatial learning and visual attention.

Finally, while it was not the intention of this study to investigate environmental familiarity, some of our participants did have varying levels of familiarity with the study area; this was because we had difficulty finding participants in health pandemic times who had never been to this area. We controlled for this factor in our models and, in doing so, observed that people more familiar with the study area showed improved performance in recognizing seen landmarks in the traversed environment, recalling route directions, and reconstructing the sequence of seen landmarks, regardless of the landmark visualization style. These findings are not surprising and align with previous work suggesting that wayfinders’ spatial knowledge formation increases with increased environmental exposure (Montello, 1998; Siegel & White, 1975). We cannot draw strong conclusions about this finding because we only had 10 people more familiar with the area than others. However, it poses an interesting future research question about the interplay between mobile map design, varying environmental familiarity of mobile map users, and respective processes of spatial knowledge formation.

## 5. Conclusions and implications

Taken together, the present real-world study found some evidence for the notion that the visualization style of landmarks on mobile map aids partially modulates wayfinders’ gaze behavior, which, in turn, can predict some aspects of spatial learning. The study highlighted that self-reported spatial abilities are a consistent predictor of several spatial learning tasks and that individuals with lower spatial abilities were more likely to benefit from landmark visualizations with greater fidelity. Our study’s primary take-home message is that mobile map design decisions, including, for example, landmark visualization style or any other relevant information visualized on the navigation aid, should be adapted to wayfinders’ individual spatial abilities, their background and training, preferences, needs, and the changing environmental context (i.e., environmental familiarity, etc.). Our study provides further insights into the role of landmarks as environmental features that scaffold wayfinders’ mental spatial representation of the environment, and shows that future map-based navigation aids should focus on a visually salient depiction of landmarks to direct the attention of wayfinders with varying spatial abilities to these features for sustained spatial learning. Hence, human-adaptive mobile maps of the future could adapt landmarks’ visual saliency dependent on wayfinders’ spatial abilities during map-aided navigation and on changing environmental contexts, such as the environmental familiarity of the navigators for mitigated navigation-related cognitive demands (Fabrikant, 2023a, 2023b).

## Data Availability

This study’s preregistration, data, and analyses (R-code) are available online at the Open Science Framework repository: https://doi.org/10.17605/OSF.IO/AKRJN.
